# Evaluating the Antimicrobial and Anti-Hemolytic Activity of Synthesized Pseudopeptide against *Leptospiral* Species: In Silico and In Vitro Approach

**DOI:** 10.3390/molecules28031106

**Published:** 2023-01-22

**Authors:** Chandan Dharmashekar, Bhargav Shreevatsa, Anisha S. Jain, Bhavana Harendra, Sushma Pradeep, Prashanth M. Vishwanath, Pranav Singh, Balamurugan V, Vinod KK, Sharanagouda S. Patil, Ali A. Shati, Mohammad Y. Alfaifi, Serag Eldin I. Elbehairi, Raghavendra G. Amachawadi, Shiva Prasad Kollur, Chandan Shivamallu

**Affiliations:** 1 Department of Biotechnology and Bioinformatics, JSS Academy of Higher Education and Research, Mysuru 570 015, India; 2 Department of Microbiology, JSS Academy of Higher Education and Research, Mysuru 570 015, India; 3 Department of Biochemistry, JSS Academy of Higher Education and Research, Mysuru 570 015, India; 4 Department of Medicine, Kasturba Medical College, Manipal Academy of Higher Education, Manipal, Udupi 576 104, India; 5 ICAR, National Institute of Veterinary Epidemiology and Disease Informatics (NIVEDI), Yelahanka, Bengaluru 560 064, India; 6 Biology Department, Faculty of Science, King Khalid University, Abha 9004, Saudi Arabia; 7 Cell Culture Lab, Egyptian Organization for Biological Products and Vaccines (VACSERA Holding Company), 51 Wezaret El-Zeraa St., Agouza, Giza 12654, Egypt; 8 Department of Clinical Sciences, College of Veterinary Medicine, Kansas State University, Manhattan, KS 66506, USA; 9 School of Physical Sciences, Amrita Vishwa Vidyapeetham, Mysuru Campus, Mysuru 570 026, India

**Keywords:** *Leptospira*, Microscopic Agglutination Test (MAT), pseudopeptide, sphingomyelin enzymes, molecular docking

## Abstract

Bacterial infections are one of the leading causes of morbidity, mortality, and healthcare complications in patients. Leptospirosis is found to be the most prevalent, re-emergent, and neglected tropical zoonotic disease worldwide. The adaptation to various environmental conditions has made *Leptospira* acquire a large genome (~4.6 Mb) and a complex outer membrane, making it unique among bacteria that mimic the symptoms of jaundice and hemorrhage. Sph2 is another important virulence factor that enhances hemolytic sphingomyelinase—capable of moving inside mitochondria—which increases the ROS level and decreases the mitochondrial membrane potential, thereby leading to cell apoptosis. In the present study, 25 suspected bovine serum samples were subjected to the Microscopic Agglutination Test (MAT) across the Mysuru region. Different samples, such as urine, serum, and aborted materials from the confirmed MAT-positive animals, were used for isolation and genomic detection by conventional PCR targeting virulence gene, Lipl32, using specific primers. Further, in vitro and in silico studies were performed on isolated cultures to assess the anti-leptospiral, anti-hemolytic, and sphingomyelinase enzyme inhibition using novel pseudopeptides. The microdilution technique (MDT) and dark field microscope (DFM) assays revealed that at a concentration of 62.5 μg/mL, the pseudopeptide inhibited 100% of the growth of *Leptospira* spp., suggesting its efficiency in the treatment of leptospirosis. The flow cytometry analyses show the potency of the pseudopeptide against sphingomyelinase enzymes using human umbilical vein endothelial cells (HUVECs). Thus, the present study demonstrated the efficacy of the pseudopeptide in the inhibition of the growth of *Leptospira*, and therefore, this can be used as an alternative drug for the treatment of leptospirosis.

## 1. Introduction

Leptospirosis, a zoonotic disease, is among several bacterial infections caused by *Leptospira interrogans*, belonging to the genus Leptospira, which comprises over 22 species under the pathogenic and saprophytic categories [1]. Recently, it has been reported that leptospiral outbreaks have increased due to human activities such as tourism within wildlife areas and army expeditions for training and other combat purposes. Globally, about 350,000–500,000 fatalities occur due to leptospirosis alone, and it is still underrated due to inadequate awareness and diagnosis. Therefore, leptospirosis-caused infections are still one of the leading zoonotic diseases, resulting in millions of morbidities worldwide [2]. It has a greater incidence in developing countries; however, is of great concern to developed countries as well due to its higher rates of incidence [3].

Leptospira is a gram-negative bacterium with rats, dogs, and cattle as its primary hosts. These primary hosts are mostly asymptomatic, with notable pathogenesis observed upon infection in humans—ranging from mild to severe symptoms [4]. They often enter the human body systems through the consumption of fecal-contaminated food and water, mainly during disasters, such as during natural calamities (floods and/or earthquakes). The disease causes mild symptoms ranging from common colds and myalgia to severe symptoms such as jaundice, hepatitis, hemorrhage, bleeding of the gastrointestinal tract, conjunctiva suffusion, myocarditis, uveitis, and others [5]. Prolonged illness and suffering ultimately lead to mortality. At the onset, the symptoms are often mild and go unnoticed, but are followed by an overwhelming relapse, causing drastic damage to the infected person in a short period [6]. More often, the symptoms of leptospirosis overlap with other common diseases such as jaundice, influenza, dengue, and Weil’s disease, and therefore, symptomatic diagnosis is inaccurate [7]; moreover, timely diagnosis is extremely difficult, ultimately leading to infection for an extended time. In turn, this delay in detection causes chronic conditions with damage to the kidney, liver, and meningitis, ultimately leading to death. Most often, benzylpenicillin and doxycycline are the standard drugs used for treatment, but they are associated with adverse effects such as anaphylaxis. Some of the *Leptospira* spp. also develop drug resistance, causing challenges in treating them efficiently [8,9]. These pathogens are also known as hemolyse erythrocytes due to the presence of unique sphingomyelinase activity. The sphingomyelinase enzymes catalyze the hydrolysis of sphingomyelin into phosphorylcholine and ceramide. Depending upon optimum pH, their enzymatic activity can be either alkaline, acidic, or neutral. Both sphingomyelinase and the hemolytic activity were expressed from a single gene, which were further designated as *sphA*. The members of sphingomyelinase exhibit a similar sequential structure found in both *S. aureus* and *Bacillus subtilis*. The members of sphingomyelinase enzymes play a crucial role in cytotoxicity towards lymphocytes and macrophages as they are an integral part of leptospirosis. These adverse effects have raised the need for an antibiotic alternative therapy for the treatment of leptospirosis. The present study focuses on the anti-leptospirosis activity of pseudopeptides, which are known for their potential antimicrobial activity, mimicking the action of naturally occurring peptides [10]. However, they have the additional benefit of enhanced stability due to the presence of modified amino acids that cannot be easily degraded by enzymes. Furthermore, the peptide bond, unlike the true bond formation between the CO-NH associated with the α-carbon atom, is bound to a carbon other than the α-carbon atom, thus rendering it less susceptible to degradation [11]. Thus, the synthesized pseudopeptides can be a great antibiotic alternative to conventional drugs as they mimic the function of natural peptides, are more biocompatible, possess greater stability and half-life than naturally occurring peptides, and can be used as an alternative drug against virulent Leptospiral species [12]. The aim of this study was to investigate the anti-leptospiral activity of the pseudopeptide-targeting sphingomyelinase enzyme both by in silico and in vitro approaches.

## 2. Results and Discussion

### 2.1. Molecular Detection of Leptospira

**Figure 1:**
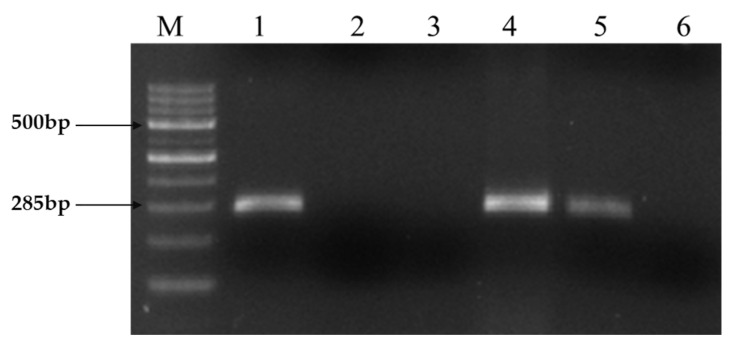
PCR amplification of Leptospiral DNA using primer sets G1/G2. Expected PCR product size of 285 bp obtained using both primer sets from strains belonging to the Ethidium bromide-stained agarose gel showing PCR products. Lane M: Molecular size marker 100 bp DNA Ladder. Lane 1: Positive control (Reference: Hardjo strain), Lane 2: Negative control (Staphylococcus Spp), Lane 3: No template control, 4: Positive isolate (KVAFSU_BANG_KAR I), 5: Positive isolate (KVAFSU_BANG_KAR II), 6: Negative sample.

The proposed 16S rRNA G1 and G2 primers have been proven to be highly specific to *L. interrogans* serovars hardjo and copenhagen, and they exclusively amplify genes in genomic DNA extracted using the phenol chloroform isoamyl alcohol method. This established that the MAT development observed on solid medium was caused by *L. interrogans* hardjo by confirming the specificity of the primers to the respective genes of certain serovars, such as hardjo and copenhagen. Since the primers were headed for amplification of metabolic genes, such as designed 16S and G1 and G2 primers, which are very necessary and unique for Leptospiral development and survival, this approach did not generate false-positive findings. The PCR amplification conditions were optimized with a gradient PCR protocol where the gradient for annealing temperature was set from 49 °C to 55 °C for 1 min based on the hypothetical Tm value that was calculated while designing a primer, along with varying concentrations of genomic DNA ranging from 5 ng to 50 ng concentrations. All the genes were found to amplify well at a 10 ng concentration of genomic DNA (Figure 1).

### 2.2. In Vitro Anti-Leptospiral Activity

**Figure 2:**
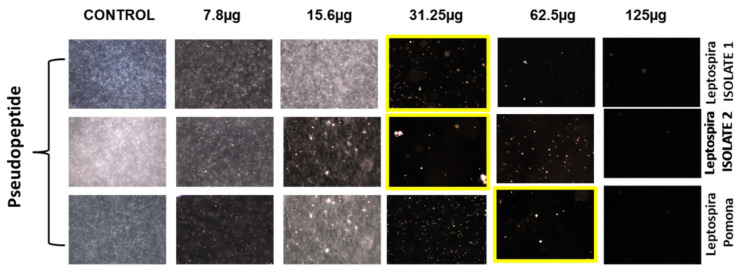
Effect of diverse concentrations of pseudopeptide against isolated *Leptospira* species by dark field microscopic technique.

The pseudopeptide was evaluated for its anti-leptospiral activity using microdilution and the dark field microscopic technique against two isolated and one standard strain, L. pamona (Figure 2). It was observed that during the microdilution test, the pseudopeptide showed good inhibitory activity for both the isolates and also at concentrations of 31.25 μg, and *L. pamona* showed inhibition at a concentration of 62.5 μg upon treatment using the pseudopeptide.

### 2.3. The Anti-Hemolytic Activity of the Pseudopeptide

**Figure 3:**
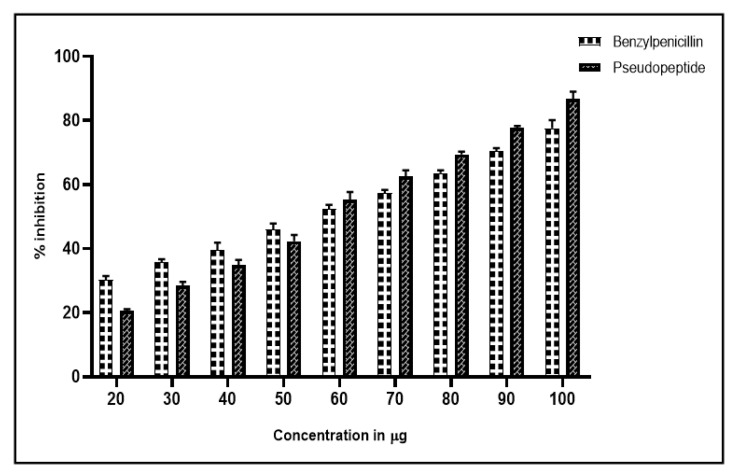
Antihemolytic profile of pseudopeptide exhibiting 30% inhibition of hemolysis at 25 µg per 100 µL concentration, with the highest inhibition of hemolysis of 85% at 100 µg per 100 µL concentration; whereas benzylpenicillin exhibits 20% inhibition of hemolysis at 25 µg per 100 µL concentration, with the highest inhibition of hemolysis of 75% at 100 µg per 100 µL concentration.

RBCs infected with *Leptospira* sp. were effectively inhibited by pseudopeptide and benzylpenicillin. While pseudopeptide exhibited 85% inhibition of hemolysis at 100 µg per 100 µL concentrations, benzylpenicillin showed 75% inhibition at the same concentration (Figure 3).

### 2.4. Sphingomyelinase Enzyme Assay

**Figure 4:**
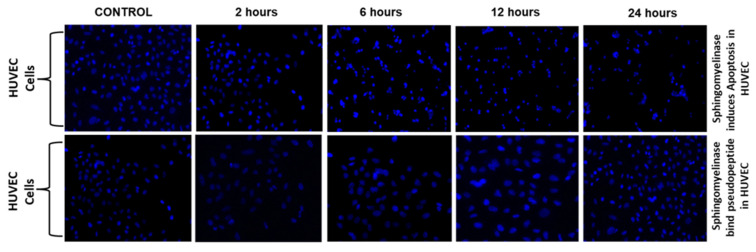
Pseudopeptide inhibition of Sph2 in HUVEC cells. Flow cytometry analysis of AnnexinV-FITC/PI-stained HUVEC cells. The cells were treated with different concentrations of pseudopeptide ranging from 250 µg to 1 mg with Sph2 for 2, 6, 12, and 24 h.

An increase of ROS and decrease of the mitochondrial membrane led to cell apoptosis. Hence, in the present study, we evaluated the mechanism of Sph2 that causes cell apoptosis in HUVEC cells. Flow cytometry was used to investigate the apoptosis levels in HUVEC cells with a combination of FITC-conjugated annexin V, pseudopeptide, and propidium iodide (PI) stain in a time-dependent manner, which shown in Figure 4. The addition of pseudopeptide resulted in a significant reduction of apoptosis in HUVEC cells from 48% to 18%. Further, Hoechst 33,342 staining was used to examine the morphological changes of cellular nuclei and cell apoptosis.

### 2.5. Protein Structure Validation and Binding Site Prediction

**Figure 5:**
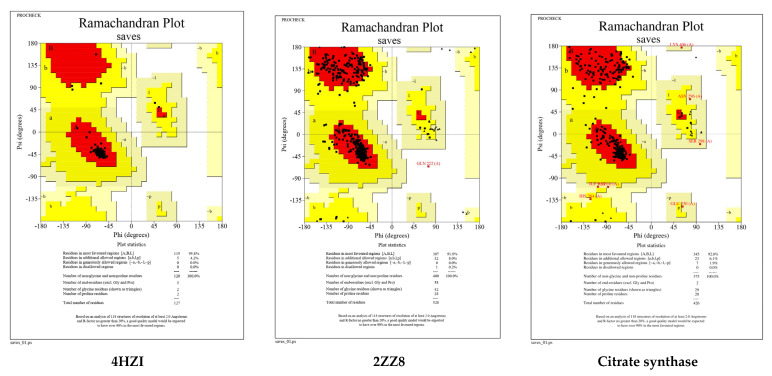
Ramachandran plots generated by PROCHECK tool. The residues are shown in the most favored (red), further allowed (yellow), liberally allowed (light yellow), and disallowed zones (white).

**Figure 6:**
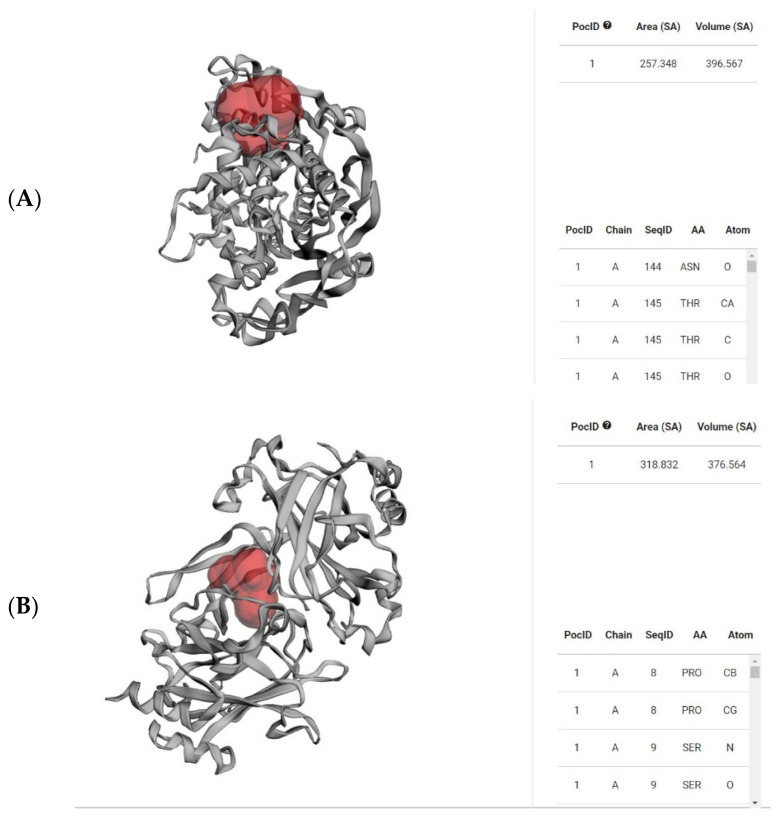
Red-colored regions determine the binding pocket of the abovementioned proteins (**A**) 4HZI, the *Leptospira interrogans* ATPase subunit of an orphan ABC transporter, and (**B**) 2ZZ8, LipL32, the most abundant surface protein of pathogenic *Leptospira* spp. predicted by CASTp.

The Ramachandran plots of the selected *Leptospira interrogans* proteins were initially checked, but initial validation revealed less than 90% of residues in the allowed region. However, after refinement, the proteins revealed considerably better percentages of residues in the allowed region, indicating that the proteins are stable enough to move forward with further molecular docking studies. The geometrical assessment of backbone psi and phi dihedral angles of all Leptospiral proteins revealed that 89.7%, 90.8%, and 91.8% of residues fell into the most favored regions of the proteins’ ATPase subunit of an orphan ABC transporter, LipL32, and citrate synthase of *Leptospira interrogans*, respectively. According to the PROCHECK results, the predicted models were of good quality. Figure 5 shows the percentage of residues in the favored, allowed, and outlier regions of all three proteins. The CASTp server revealed all surface pockets in protein structures, together with their exact volume and area, as well as detailed information on all atoms involved in their formation. (Figure 6)

### 2.6. Molecular Docking Studies

In order to identify the mode of action of the designed pseudopeptide, its interactions with some of the important Leptospiral proteins for cell survival were evaluated. The findings of the present study revealed that the pseudopeptide showed a potential inhibition against all three selected *Leptospira interrogans* proteins.

#### 2.6.1. Synthesized Pseudopeptides Interacting with 4HZI Protein

**Figure 7:**
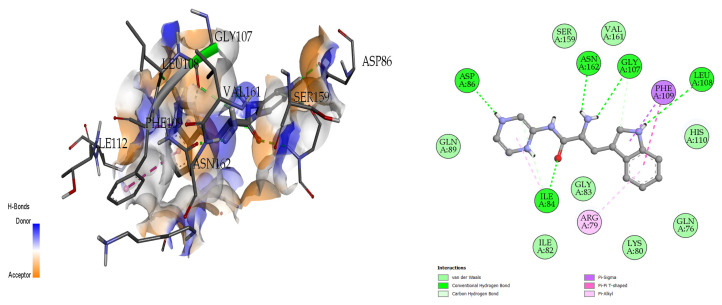
Three-dimensional (**left**) and two-dimensional (**right**) docking visualization of 4HZI protein with pseudopeptide.

The docking analysis and visualization of the 4HZI protein are shown with the pseudopeptide. The pseudopeptide shows good binding affinity (−8 Kcal/mol) and ASP-86, ILE-84, ASN-162, GLY-107, and LEU-108 were the amino acids involved in the docking interaction at the binding pocket of the 4HZI protein, which are shown in Figure 7.

#### 2.6.2. Synthesized Pseudopeptides Interacting with 2ZZ8 Protein

**Figure 8:**
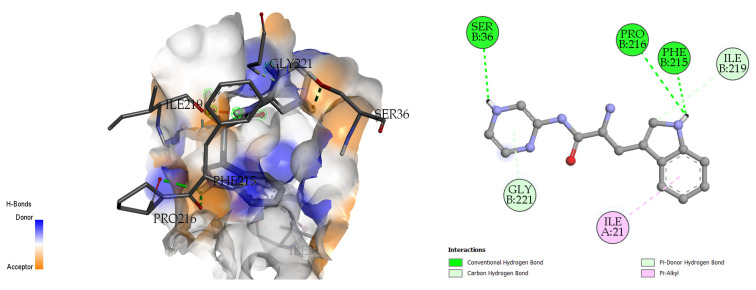
Three-dimensional (**left**) and two-dimensional (**right**) docking visualization of 2ZZ8 protein with pseudopeptide.

The docking analysis and visualization of the 2ZZ8 protein with pseudopeptide are shown in Figure 8. The pseudopeptide showed a comparatively greater binding affinity of −6.9 Kcal/mol. The amino acids that were involved in the docking interaction at the binding pocket of the 2ZZ8 protein include SER-36, PRO-216, and PHE-215.

#### 2.6.3. Synthesized Pseudopeptides Interacting with Citrate Synthase of Leptospira Interrogans

**Figure 9:**
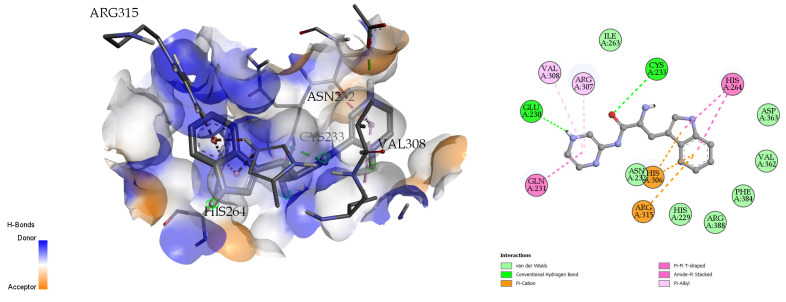
Three-dimensional (**left**) and two-dimensional (**right**) docking visualization of citrate synthase protein with pseudopeptide.

The docking analysis and visualization of the citrate synthase protein with pseudopeptide were shown in Figure 9. The docking analysis estimated a good binding affinity (−7.7 Kcal/mol), and the amino acids involved in the docking interaction at the binding pocket of the citrate synthase protein include GLU-230, CYS-233, HIS-306, and ARG-315.

### 2.7. Molecular Dynamics Simulation

**Figure 10:**
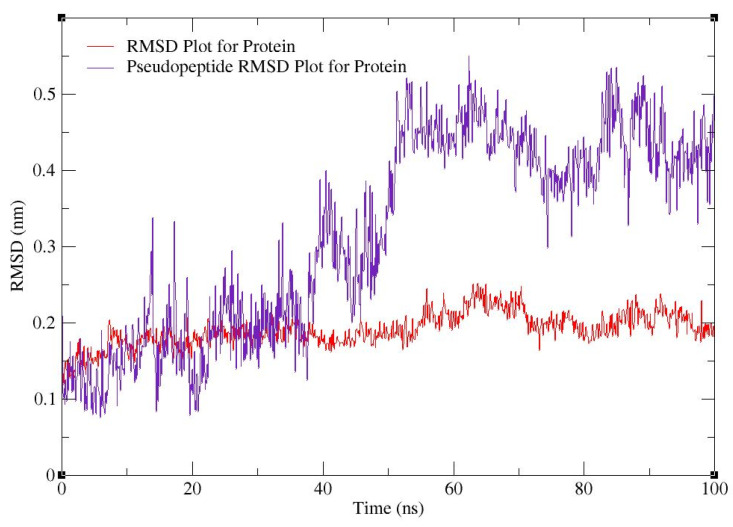
RMSD plot for 4HZI protein (red) and pseudopeptide–protein docked complex (purple).

**Figure 11:**
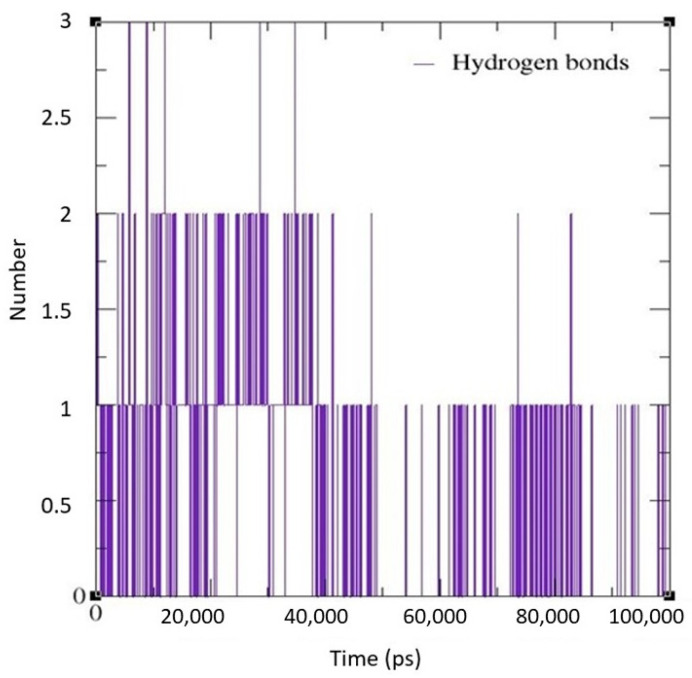
H-bond graph predicted using the GROMACS MD simulation software for the docked complex.

For MD simulations, pseudopeptide docked with 4HZI, the *Leptospira interrogans* ATPase subunit of an orphan ABC transporter that was considered, as it showed the highest binding affinity when compared to other proteins. In this study, the CHARMM36 all-atom force field of GROMACS 2021 was used to perform MD simulations for the pseudopeptides to evaluate their binding interactions with the target protein. A total of 100 ns MD simulations were run for the complex. Figure 10 shows the root square mean deviation (RMSD) of the docked complex over the simulation time. The RMSD of the protein backbone with respect to the starting conformation versus simulation time were displayed to examine the global behavior of the tested systems. The RMSD of the pseudopeptide gradually increased and stabilized at 50 ns. Smaller fluctuations were observed for the pseudopeptide RMSD graph. Using 100 ns simulation trajectories, the number of H-bonds between ligand and protein was investigated (Figure 11). The docked complex of the pseudopeptide and *Leptospira interrogans* ATPase subunit of an orphan ABC transporter indicated three hydrogen bonds, at most, with an average of one hydrogen bond throughout the simulation time.

## 3. Material and Methods

### 3.1. Isolation and Identification of Leptospira Isolates

Isolation and identification of Leptospira isolates have been carried out in different samples, such as humans and domestic animals—including cattle and pigs—as well as different environments such as natural landscapes, agricultural fields, rural settlements, and urban areas. The isolation procedure was followed as per the method given by Narkkul et al. [, with minor modifications. Briefly, the sample collected from the serum/blood/urine/tissue were inoculated (1–2 drops) directly into the selective media, called EMJH (Ellinghausen and McCullough, modified by Johnson and Harris) medium, which contains 500 μg of 5-fluorouracil per mL at room temperature. After the incubation period of 14 days, the cultures were filtered through a 0.2 µm membrane filter and sub-cultured periodically from 8 to 10 weeks. After successful isolation, the cultures were stored in EMJH medium until further use and sub-cultured periodically to maintain the viability of the organism [13].

### 3.2. Synthesis of Pseudopeptide

**Figure 12:**
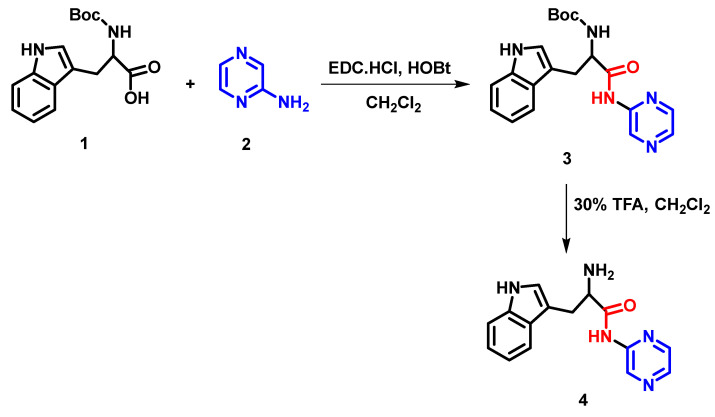
Schematic representation showing the chemical synthesis of pseudopeptide.

The pseudopeptide was synthesized using the previously reported method [8]. A general overview of the synthetic route of the pseudopeptide under study is depicted in Figure 12.

### 3.3. Polymerase Chain Reaction (PCR)

The Leptospira isolates were subjected to genomic DNA extraction using a QIAmp DNA mini kit (Qiagen, Valencia, CA, USA) as per the manufacturer’s instructions. PCR was performed using G1 and G2 primers (G1 5′-CTG AAT CGC TGT ATA AAA GT 3′ and G2 5′-GGA AAA CAA ATG GTC GGA AG-3′) that targeted Leptospira genus-specific *secY* gene (ACD46818.1) methodology described elsewhere Meenambigai et al [14]. DNA of *L. interrogans* serovar Hardjo and *Staphylococcus* spp. were used in PCR as positive and negative controls, respectively.

### 3.4. In Vitro Anti-Leptospiral Activity

The in vitro anti-leptospiral activity by microdilution and dark field microscope assays was performed with 96-well microtiter plates as per the previously published procedure [15]. Briefly, each plate included a positive control (bacteria), negative control (EMJH media only), and synthesized pseudopeptide with varying concentrations ranging from 25, 50, 75, 100, 125, and 150 μg/mL, while benzylpenicillin (25 μg/mL) was used as the standard control drug. One-hundred microliters of leptospiral inoculum containing 2 × 10^6^ leptospiral organism per ml were added to 96-well plates to increase the final volume to 200 µL and the plates were incubated in 30 °C. After three days of incubation, 20 µL of 10-times concentrated alamarBlue was added to all wells. No change of color from dark blue to pink shows the potency of pseudopeptides at different concentrations and were compared with a standard drug, benzyl penicillin. Furthermore, the viable cells were treated with varying concentrations (5 μL each tube) of the test sample in a microtiter plate. The plates were mixed thoroughly by covering it with aluminum foil and incubated for 30 min at room temperature. The organism was then observed under the dark field microscope to assess the extent of inhibition, and the obtained results were tabulated as MIC (Table 1).

### 3.5. Hemolytic Activity

The effects of the pseudopeptide on hemolysis were assessed in vitro, which is one of the many symptoms of leptospirosis. The assay was performed as per the previously published procedure [16]. Briefly, 5 mL of blood was collected into a tube containing trisodium citrate (anticoagulant) from healthy volunteers not under non-steroidal anti-inflammatory drug (NSAIDs) administration up to two weeks before the blood collection. This was subjected to centrifugation for 5 min at 3000 rpm to separate plasma and RBCs. The erythrocytes were collected by discarding the supernatant, which was then washed thrice using phosphate buttered saline. The pellet was further suspended in the same buffer (1:9 dilution) and used within 6 h. Aliquots of 50 µL of erythrocytes were taken in four tubes, treated with 25 µL of each of the five Leptospira strains, and incubated at room temperature for 30 min along with triton-X100 as a positive control to demonstrate 100% hemolysis. This incubation facilitates the *Leptospira* sp. to infect the erythrocytes. In the post-incubation period, 100 µL of varying concentrations (25, 50, 75, 100, 125, and 150 µg) of the pseudopeptide were added to all the tubes, including the control. The standard drugs, penicillin and benzylpenicillin (50 µg/mL), were taken as standards for the experiment with the same concentration as that of the pseudopeptide. The tubes were then incubated in a water bath for 60 min at 37 °C. Furthermore, the tubes were subjected to centrifugation at 3000 rpm for 3 min, and the supernatant was collected. The optical density of the supernatant was evaluated at 540 nm with an erythrocyte- and phosphate-buffered saline as a blank.

### 3.6. Cell Lines and Cultivation

Human vascular endothelial cell lines derived from umbilical cord vein, HUVEC cells, were procured from American Type Culture Collection (ATCC CRL-4053). The cells were cultured at 37 °C in RPMI 1640 media, 10% fetal bovine serum, and streptomycin and penicillin (100 μg/mL) antibiotics in a 5% CO_2_ incubator.

### 3.7. Sphingomyelinase Enzyme Assay

The activity was performed by coupled assay using the Sphingomyelinase Assay Kit (ThermoFischer Scientific, Amplex™,TX, USA) as per the manufacturer’s instructions. The HUVEC cell lines were infected with *L. interrogans* as described previously by Jin et al [17]. To the infected cells, 150 µM of pseudopeptides labelled with FITC were added and incubated at 37 °C for about 24 h. After incubation, the cell culture supernatant was discarded and the intermediate-adhering leptospires were collected for the Sphingomyelinase assay. The Sphingomyelinase assay was performed in flat bottom black polystyrene 96-well microtiter plates. The reaction mixture contains 100 μL of test sample and 100 μL of μM Amplex red reagent (composed of 0.2 U/mL choline oxidase, 2 U/mL horseradish peroxidase, 0.5 mM sphingomyelin, and 8 U/mL alkaline phosphatase) and was incubated at 37 °C for about 90 min. The obtained fluorescence color was measured both in excitation and emission state with wavelengths ranging from 530 nm to 590 nm using Thermo Multiskan FC Microplate Reader. The reaction buffer without sphingomyelinase was used as a negative control. The assay was repeated with three biological replicates.

### 3.8. In Silico Analysis

#### 3.8.1. Protein Structure Preparation and Validation

**Figure 13:**
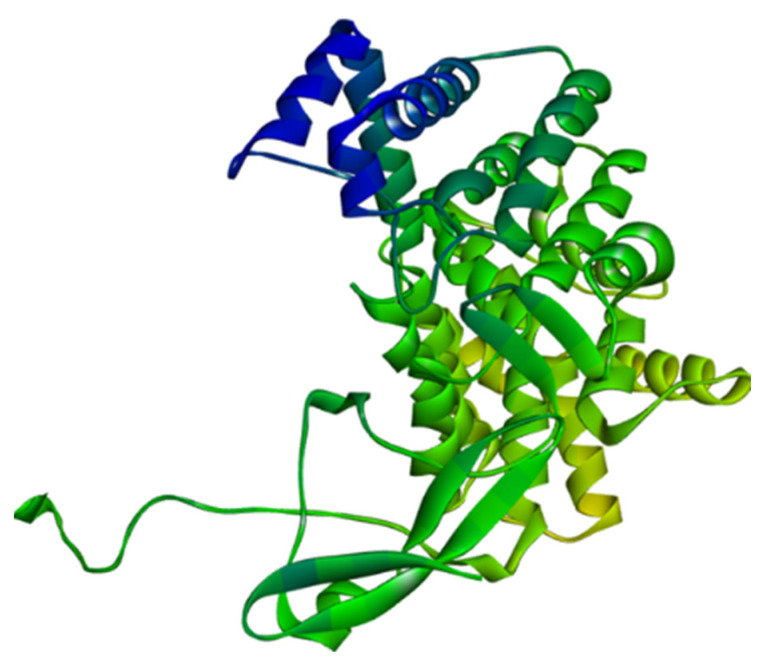
Homology modelled structure of citrate synthase of Leptospira interrogans using SWISS-Model tool.

For the in silico study, three Leptospiral metabolic proteins were chosen. The structure of citrate synthase was modeled using homology modelling by the SWISS-MODEL server [18], which is accessible via the Expasy web server and relies on the target and template proteins’ evolutionary relationship. The sequence for modelling this protein was obtained from NCBI under the accession number, WP_061270692. A search for homologous proteins in a library of experimentally known protein structures was used to identify potential structural templates. The structures of the other two Leptospiral proteins, the ATPase subunit of an orphan ABC transporter and LipL32 and the most abundant surface proteins of pathogenic *Leptospira* spp., were taken from the RCSB Protein Data Bank [19] with the PDB ID 4HZI [20] and 2ZZ8, each possessing a structure resolution of 1.85 Å and 2.01 Å, respectively (Figure 13).

**Figure 14:**
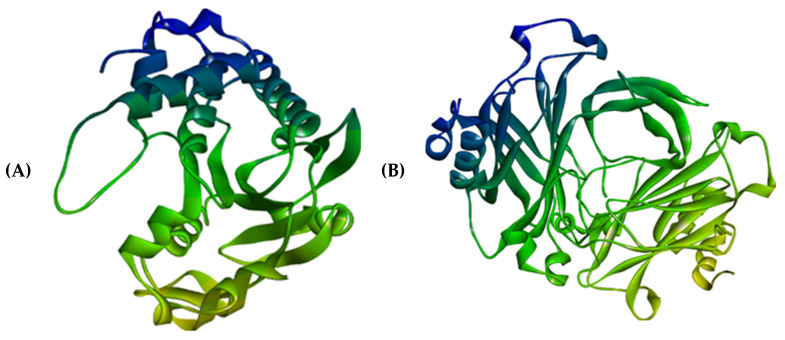
Three-dimensional structures of (**A**) 4HZI, the Leptospira interrogan’s ATPase subunit of an orphan ABC transporter and (**B**) 2ZZ8, LipL32, the most abundant surface protein of pathogenic *Leptospira* spp.

The selected protein structures were refined using the ModRefiner tool, which is accessible via the Zhang Lab web server. Using the PROCEHCK program from the online server, UCLA-DOE LAB—SAVES v6.0, the abovementioned protein structures were confirmed and evaluated for precision based on the Ramachandran plot. The proteins’ binding sites were identified using the CASTp (Computed Atlas of Surface Topography of Proteins 3.0) tool [21]. In a protein structure, it provides all inner cavities and surface pockets (Figure 14).

#### 3.8.2. Ligand Preparation

**Figure 15:**
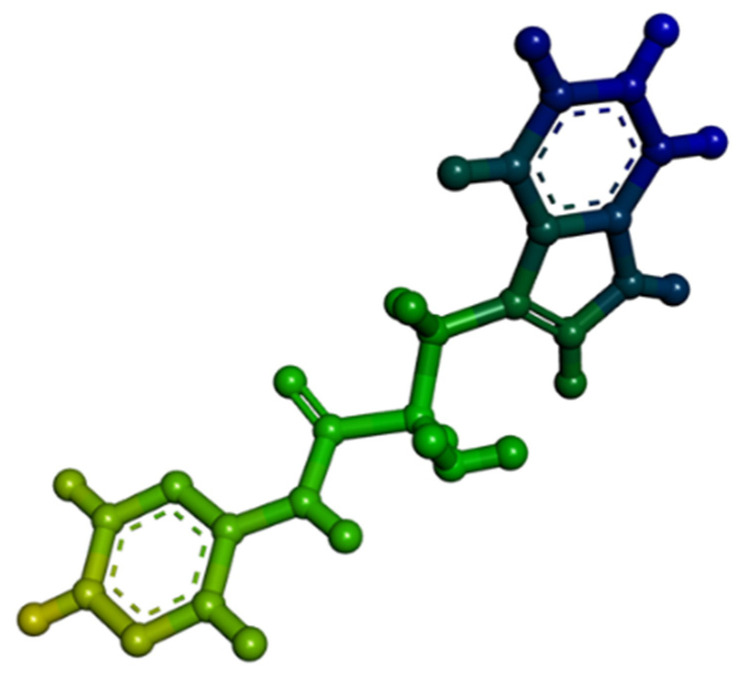
Three-dimensional structure of organic synthesized pseudopeptide.

H-NMR studies were used to predict the structure of the synthesized pseudopeptide. For in silico molecular docking and molecular simulation investigations, this structure was sketched using the ChemSketch 12.0 software. To obtain the pdb file, which is the needed file format for docking studies, file format conversion was performed using the OpenBabel GUI 2.4.1 [22]. BIOVIA Discovery Studio Visualizer 2020 [23] was used to fix up the geometry of the sketched ligand (Figure 15). The visualization software, UCSF Chimera 1.15 [24], was used to perform the energy minimization.

#### 3.8.3. Molecular Docking and Visualization

The validated protein structures were considered as the target structures for molecular docking analysis. The synthesized pseudopeptide was docked against the target leptospiral proteins using the PyRx 0.8 [25] virtual screening tool; it aims to anticipate the possible binding modalities of the three-dimensional structure of the complex based on the binding capabilities of the pseudopeptide and the target protein [26,27]. The pseudopeptide was docked using a genetic algorithm, in which the ligand undergoes conformational changes to determine the lowest energy conformation, which is the most stable structure and the most likely structure observed in in vivo systems. The best docking structure with the lowest binding energy (Kcal/mol) was chosen. To determine the non-bonded interactions, the docking complexes formed in molecular docking were further visualized using the visualization tools, Chimera and BIOVIA Discovery Studio Visualizer.

#### 3.8.4. Molecular Dynamics (MD) Simulations

After docking, an MD simulation was used to improve the final structures, examine the stability of distinct complexes, and account for solvent effects and more accurate binding energy calculations. The GROMACS 2021 was chosen as the best docked protein–peptide complex, and it was chosen to examine the dynamic binding interactions of the pseudopeptide with the target Leptospiral proteins. The NVT ensemble was performed first, with the number of molecules (N), volume (V), and temperature (T) all maintained at a constant, followed by the NPT ensemble, with the number of molecules (N), pressure (P), and temperature (T) all kept constant. Finally, 100 ns MD simulations of the protein–peptide complex were performed at 310 K and 1 bar atmospheric pressure. QtGrace was used to plot the graphs from the resulting xvg files [28,29].

## 4. Discussion

Leptospira infections are usually treated with the antibiotics, tetracyclines. Knowing how to treat leptospirosis with antibiotics requires in vivo clinical testing, which is typically done on patients who have severe and recent Leptospira infections. Earlier studies have examined the effectiveness and workings of novel chemotherapeutic compounds. This study is the first to demonstrate the effectiveness of tigecycline against *Leptospira* species in vitro. In the present study, we have designed ligands based on their core structures, which is unlike that of the terpenoid derivatives analyzed by de Oliveira et al. [30], which makes this study a novel approach to the treatment and eradication of *L. interrogans*. Several studies have reported on the anti-leptospiral activity of synthetic compounds that target various pathways for bacterial growth. Umamaheshwari et al. [31] virtually screened for various drug attributes by targeting the ATP-dependent MurD protein responsible for peptidoglycan biosynthesis. Similarly, an in silico approach for the design of anti-leptospiral molecules was carried out by Wolkmer et al. [32], who identified sodium curcuminate and Gemichalcone B as the most likely compounds that are effective against inhibiting pathways such as NF-kB and MAPK and their corresponding chemokines responsible for tubular inflammation. However, these studies did not elucidate the precise pathway and evaluate the potential of the drug attribute against pathogenic growth. In the present study, the synthetic pseudopeptide was evaluated against the growth of different serotypes of *Leptospira*, revealing promising results. Fernandes et al. [33] proved that non-receptor serine/threonine kinase p38 is essential for tumour progression, cell proliferation, and cell differentiation; moreover, increased levels of p38 increase the cytokine levels, which promotes inflammation and the development of tumors. As a result, the protein, p38, was selected as a target for avoiding the spread of inflammatory cancers. The development measures helped in our decision to use human p38 as a drug target for the development of herbal remedies for this disorder. Leptospira OMPs may cause tubulointerstitial nephritis and necrosis through a pathway that is based on Toll-like receptors (TLRs) [34]. The findings suggest that the designed pseudopeptide could be used as a potential anti-leptospiral agent after further studies on its toxicity, bioavailability, and kinetics. Molecular docking forms an essential tool in the discovery and design of new drugs [35]. It is a default process to identify and analyze docking introduction in drug discovery and forms the basis for the design and analysis of novel drugs against a specific target protein [36]. Several studies have reported the use of molecular docking to understand the probable mechanism of action for various drugs, including antimicrobial agents [37]. The degree of protein–ligand complex interaction is commonly referred to as the binding affinity in molecular docking investigations. The ligand’s ability to bind to the target depends on its affinity. The pseudopeptide showed the highest binding affinities and lowest binding energies for the chosen target proteins [38]. The target proteins had binding energies between −8 Kcal/mol and −6.9 kcal/mol, and at least three to five hydrogen bonds were formed. The current study concludes that pseudopeptides have an effective anti-leptospiral activity based on the binding energy obtained, regardless of whether the interactions between the target and ligand are bonded or non-bonded.

## 5. Conclusions

This current work focuses on the inhibition of metabolism in Leptospira to inhibit its growth and propagation instead of inhibiting the protein synthesis, as is the case for conventional antibiotics. This approach not only inhibits the growth of Leptospira, but also instantly kills the organism. Conventional antibiotics take a longer time to act on Leptospiral inhibition and their effect can be overcome by Leptospira with the help of drug resistance proteins such as multidrug efflux protein, which make the drug ineffective. However, in the case of the metabolic inhibition approach, the organism does not have enough time to cope and develop a resistance mechanism, thereby reducing the chance for development of resistance against the inhibitory molecule. Interestingly, our study has provided remarkable evidence for the efficiency of synthesized pseudopeptides in inhibiting the growth of *Leptospira*. The in vitro efficacy evaluated using the MDT and DFM assays revealed that the pseudopeptides were effective against isolated leptospiral pathogens, which was further supported by the in silico evaluation of *Leptospiral* metabolic proteins. The promising results from this study suggest that this pseudopeptide could be further evaluated in vivo in order to check for its bioavailability and kinetics for usage as an anti-leptospiral drug.

## Figures and Tables

**Table 1:** MIC of pseudopeptide against isolated *Leptospira* species by microdilution technique (MDT).

Genotype/Strain	MIC(µg/mL)	MIC(µg/mL)
*L. interrogans*/*pomona*	6.8/6.8	58.4/37.8
Isolate 1	6.8/6.8	58.4/37.8
Isolate 2	6.8/6.8	58.4/37.8
MIC(µg/mL)	6.8	58.4

Broth microdilution (micro) MICs (run 1/run 2 for each) are given in micrograms per milliliter (units per milliliter for penicillin G).

## Data Availability

Not applicable.
